# The Effects of Vitamin D Supplementation in COVID-19 Patients: A Systematic Review

**DOI:** 10.3390/ijms232012424

**Published:** 2022-10-17

**Authors:** Ángela Feiner Solís, Ana Avedillo Salas, María José Luesma Bartolomé, Sonia Santander Ballestín

**Affiliations:** 1 Department of Pharmacology, Physiology and Legal and Forensic Medicine, Faculty of Medicine, University of Zaragoza, 50009 Zaragoza, Spain; 2 Department of Human Anatomy and Histology, Faculty of Medicine, University of Zaragoza, 50009 Zaragoza, Spain

**Keywords:** vitamin D, SARS-CoV-2, vitamin D deficiency, supplementation, immune system

## Abstract

Vitamin D has an immune-modulating effect on respiratory tract infections. For this reason, it has been proposed as part of the treatment in COVID-19. Furthermore, vitamin D deficiency has been associated with worse clinical outcomes of this disease. The aim of this systematic review was to determine whether vitamin D supplementation modifies the disease course. Therefore, eleven studies involving randomised clinical trials are analysed, in which groups of COVID-19 patients with or without vitamin D supplementation as part of the treatment are compared. A control group was treated with best available therapy, and in some of the clinical trials, also with a placebo. According to the outcomes, it seems that patients benefit from receiving a daily or maintained in time vitamin D dose regardless of vitamin D serum levels at the beginning of the trial. The administration of a single vitamin D dose does not seem to have any effect on the health status of these patients. However, the outcomes are heterogeneous and larger clinical trials are necessary.

## 1. Introduction

### 1.1. Background

SARS-CoV-2 disease (COVID-19), which has spread globally, affects millions of people, and research into effective treatments in parallel with vaccine development is essential. Since the mid-1980s, vitamin D has been of particular interest due to the discovery of its ability to prevent disease, as it intervenes in mechanisms that modify the innate and adaptive immune response [1]. Indeed, vitamin D has a protective effect against respiratory tract infections [2,3], and vitamin D supplementation has been shown to reduce the incidence of respiratory infections [4]. For this reason, vitamin D has been proposed as a treatment for COVID-19. Vitamin D is represented by two liposoluble compounds: vitamin D_2_ and vitamin D_3._ Vitamin D comes mainly from exposure of the skin to sunlight and only a small percentage from the diet. Vitamin D is transported to the liver, where it is hydroxylated to the major circulating form of vitamin D (cholecalciferol or 25(OH)D3) by at least one cytochrome P450 (CYP) hydroxylase. After the transportation of 25(OH)D3 to the kidney, mediated by another CYP hydroxylase, the hormonally active form of vitamin D (1,25(OH)2D3) is synthesised. The liver converts cholecalciferol to 25-OH cholecalciferol (calcifediol). This, in turn, is a precursor of the active form, 1,25-dihydroxyvitamin D₃ (1,25(OH)2D3), also called calcitriol. Active vitamin D exerts its effects through binding to its receptor, which is widely distributed in various tissues of the body. In addition, many of them have the vitamin D-activating enzyme 25-Hydroxyvitamin D_3_ 1alpha-hydroxylase (CYP27B1), which is capable of producing 1,25(OH)2D3 from its circulating precursors [5].

### 1.2. Role of Vitamin D in SARS-CoV-2 Infection

In COVID-19, caused by the infection of the SARS-CoV-2 virus, patients who develop severe pneumonia correlate with a hyperinflammatory state resulting from a strong activation of several immune cells, leading to the uncontrolled release of large amounts of pro-inflammatory cytokines, a process known as a “cytokine storm”. These are responsible for the massive migration of inflammatory cells into the lung tissue and the release of more cytokines, proteases, free radicals and nitric oxide, which severely damages the microvascular and alveolar barrier, resulting in hyaline membrane formation, excess extravasation and alveolar oedema. This results in impaired gas exchange and consequently respiratory distress and lower arterial oxygen saturation, leading to acute respiratory distress syndrome (ARDS) [6]. A potential role of vitamin D in modulating this phenomenon has been observed. The airway epithelium and alveolar macrophages are capable of expressing the enzyme CYP27B1 and the vitamin D receptor. In other viruses and respiratory pathogens, the activation of the innate immune system leading to the increased local production of 1,25(OH)2D3 has been shown to enhance viral neutralisation and clearance while modulating the subsequent pro-inflammatory response [7].

### 1.3. Mechanism of Action of Vitamin D with Regard to Its Immunomodulatory Role

Calcitriol is essential in the regulation of phosphocalcium metabolism for proper bone formation. It also has an important immunomodulatory role on various immune cells, such as monocytes, macrophages, dendritic cells and T and B lymphocytes (8). All of these cells express the vitamin D receptor (VDR) and the enzyme CYP27B1, indicating that they are capable of producing and responding to activated vitamin D [7,8,9]. In the following, we will attempt to summarise the main actions of vitamin D on the immune system.

**Figure 1:**
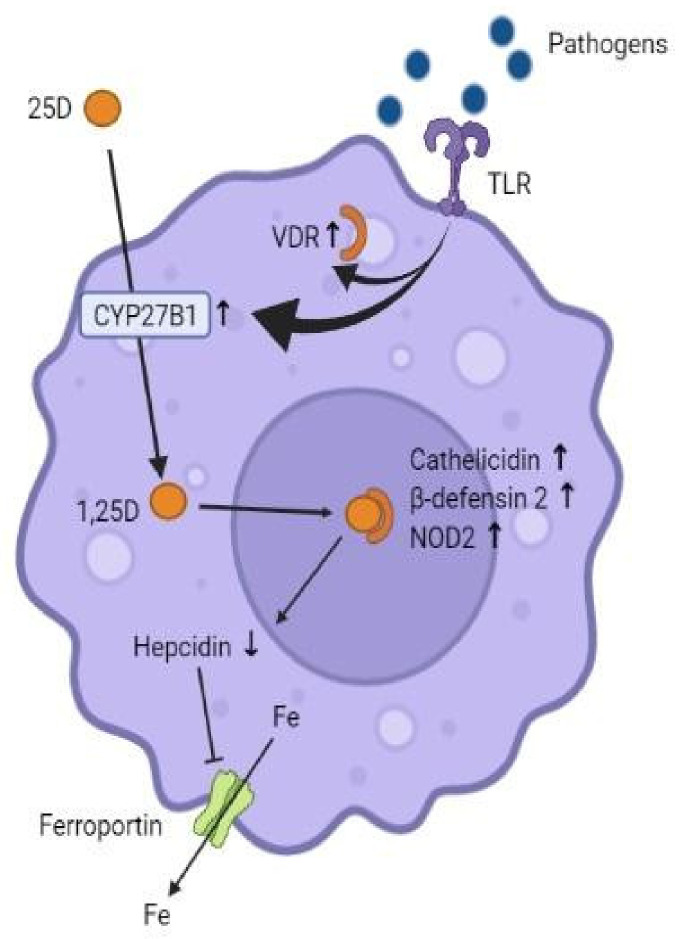
Effects of vitamin D on immune cells. Activation of toll-like receptors (TLRs) by pathogens increases the expression of VDR and CYP27B1. 1,25(OH)2D3 binds to VDR, which induces the formation of cathelicidin, β-defensin 2 and NOD2. Vitamin D suppresses hepcidin, which facilitates the outflow of intracellular iron (Fe).

Vitamin D modulates the innate immune system at several levels. In monocytes and macrophages, calcifediol is converted intracellularly by CYP27B1 to 1,25(OH)2D3. This stimulates the production of the antimicrobial peptides cathelicidin, β-defensin 2 and nucleotide-binding oligomerisation domain-containing protein 2 (NOD2), whose antimicrobial effect occurs by the destruction of bacterial and viral cell membranes or by the activation of antibiotic signalling cascades in infected cells [9]. In addition, vitamin D exerts an antimicrobial role in that it is a potent suppressor of hepcidin, which restricts the transcellular export of iron via ferroportin. In this way, 1,25(OH)2D3 enhances activity at the ferroportin level, thus facilitating the outflow of iron from the cell, leading to a reduction in intracellular iron, which is necessary for bacterial survival [7] (Figure 1). On the other hand, an essential mechanism by which cells fight viruses is autophagy in macrophages. It involves the encapsulation of viral particles for lysosomal degradation and subsequent antigenic presentation and initiation of the adaptive antiviral response [10]. Vitamin D induces autophagy by inhibiting the mTOR metabolic pathway (which inhibits autophagy) and promotes the expression of Beclin-1 and phosphatidylinositol 3-kinase catalytic subunit type 3 (PI3KC3), key enzymes for the autophagy process. The vitamin D-regulated increase in intracellular calcium and nitric oxide (NO) also stimulates PI3KC3 activity [7].

As for the adaptive immune response, T cells interact with antigen-presenting cells to induce an antigen-specific immune response [9]. At this level, 1,25(OH)2D3 reduces CD4+ T cell differentiation to Th1 and Th17 in favour of differentiation to Th2 and Treg, resulting in the decreased production of pro-inflammatory cytokines (IL1, IL6, IL12, TNFα, IL17 and IFN-γ) and increased production of anti-inflammatory ones (IL10) [11]. It follows, therefore, that vitamin D, in general, exerts an inhibitory, anti-inflammatory action on the adaptive immune system [7].

In summary, these data indicate that 1,25(OH)2D3 plays an important role in the maintenance of immune homeostasis [8] and reduces the proinflammatory state, which, in turn, is key in the pathophysiology of ARDS in SARS-CoV-2 infection [7]. In addition, as mentioned above, it induces the production of antimicrobial peptides and autophagy. Moreover, ways have been described by which vitamin D promotes gap protein and tight junction protein expression, which contributes to maintaining the integrity between lung epithelial cells, preventing virus penetration [11]. Finally, vitamin D increases the expression of angiotensin-converting enzyme 2 (ACE2), which has protective effects against inflammation, and suppresses the renin–angiotensin system [9]. ACE2 acts as a receptor for SARS-CoV-2 that allows its entry into the host cell, and SARS-CoV-2 infection leads to a reduction in its expression, which triggers an inflammatory chain reaction [12]. Furthermore, vitamin D’s induction of ACE2 increases the soluble ACE2, and it has been suggested that soluble ACE2 binding to SARS-CoV-2 prevents it from binding to membrane-bound ACE2 [13]. Because of all of these mechanisms, it is postulated that vitamin D may be protective in this disease.

### 1.4. Vitamin D Deficiency—A Risk Factor in COVID-19?

On several occasions, vitamin D deficiency has been shown to be a general risk factor in the development of acute respiratory disease [14]. In the current pandemic context, there have been several observational studies showing a worse course of COVID-19 in patients with low baseline serum calcifediol levels [15,16,17,18,19,20,21,22]. Other facts that reinforce the role of this vitamin as protective against COVID-19 include that most outbreaks occur in winter, when calcifediol is at its lowest levels and the number of cases reported in the Southern Hemisphere in late summer is low; the case fatality rate also increases with age and chronic disease comorbidity, both associated with lower calcifediol levels [23]. However, the risk factors for developing severe COVID-19 are the same as those for developing vitamin D deficiency (age, obesity, black or Asian ethnicity, poor general health, poor diet and comorbidities such as diabetes, liver and kidney disease), so it is difficult to determine whether vitamin D deficiency is itself a risk factor in the development of severe COVID-19 [24,25]. Although this overlap is controversial and needs to be studied, it could be explained by the simple fact that the healthier population spends more time outdoors and eats more healthily compared to the less healthy [14]. Applying the Bradford–Hill criteria, there is a causal association between low vitamin D status and COVID-19 infection and disease [26], but the question of whether vitamin D is a marker of poor disease prognosis or a possible risk factor with beneficial effects from supplementation remains to be answered [27].

### 1.5. Justification and Aim

Several meta-analyses and randomised clinical trials have shown the protective effects of vitamin D against acute respiratory infections [2,3]; however, the impact of vitamin D in the treatment of COVID-19 is still controversial [9]. For this reason, this systematic review focuses on the review of randomised clinical trials that have been conducted so far, as the interest lies in knowing whether active vitamin D supplementation in infected patients leads to an improvement in the course of the disease and should therefore be considered as part of the treatment.

The main objective of this study is to determine the effects of vitamin D supplementation on parameters reflecting the health status of patients affected by COVID-19: muscle condition, need for admission to the intensive care unit (ICU), mortality, length of hospitalisation, inflammatory markers, arterial O_2_ saturation, etc. The secondary objective is to observe whether there are differences in the effect of this supplementation according to baseline levels of calcifediol.

## 2. Methodology

### 2.1. Search Strategy

A systematic review of the published scientific literature on the effects of vitamin D supplementation in patients with active COVID-19 was conducted.

The search was made using PubMed, Web of Science (WOS) and the Cochrane COVID-19 Study Register, which also includes trials published in ClinicalTrials.gov-COVID-19 subset and the WHO International Clinical Trials Registry Platform (ICTRP).

The studies were identified by combining the name “COVID-19” and “SARS-CoV-2” with “vitamin D”. MeSH (medical subject heading) terms and the Boolean operator “AND’’ were also used in the search.

Thus, to specify that articles associated with this topic were included, but that they were only experimental studies, the following strategy was followed: in PubMed we searched for COVID-19 AND “vitamin d”, and then filtered by type of article, selecting the Clinical Trial and Randomized Controlled Trial options; in the Cochrane COVID-19 Study Register, we included the same combination of terms and then included the Report Results filters, so that only those studies that provided results would appear, and in terms of study characteristics, we selected Interventional. Finally, in Web of Science, the combination of terms that allowed us to obtain the most appropriate results was ((ALL = (COVID-19 AND “vitamin d”)) AND ALL = (“clinical trial”)) NOT ALL = (“systematic review”).

**Figure 2:**
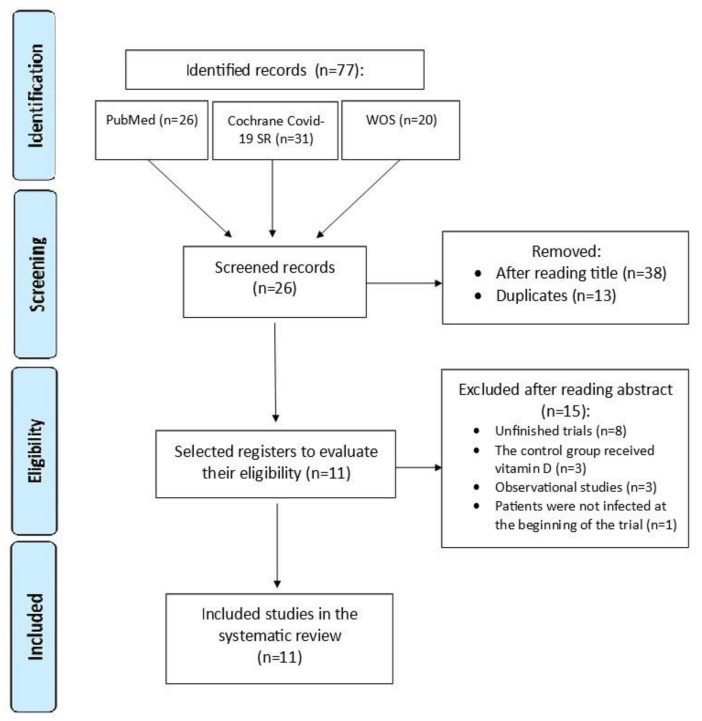
PRISMA flow chart at four levels.

The guidelines of the PRISMA (Preferred Reporting Items for Systematic Reviews and Meta-Analyses) statement [28,29] for the correct conduct of systematic reviews were followed. Articles published before May 2022 were retrieved. The research question was constructed using the PICO strategy. The inclusion and exclusion criteria were defined prior to article selection. After removing duplicates, the titles and abstracts were screened, excluding those that did not meet the inclusion criteria. The remaining records were then assessed for eligibility by careful review of their full texts. A flow chart illustrating the study selection process is shown in Figure 2.

### 2.2. Inclusion Criteria

Regarding the types of studies, clinical trials and randomised controlled trials (RCTs) were included. The studies were completed and included the results. Both the study and comparison groups had COVID-19 at the time of the study or were newly diagnosed with COVID-19. The rest of the inclusion criteria were proposed according to the PICO algorithm (Table 1).

We decided to conduct this review only on the basis of randomised clinical trials, on the one hand because they are subject to a very high degree of scientific evidence, and on the other, because they are the best way to answer the question posed by this review: whether or not the health status of patients infected with COVID-19 improves with vitamin D administration. However, in order to obtain more evident results, we preferred to include only those trials in which the control group did not receive specific vitamin D supplements, so we excluded those in which two groups receiving different doses of vitamin D were compared. It was thought necessary to first answer the question of whether vitamin D supplementation is a treatment option or not; effective doses will need to be studied once this fundamental question has been answered. Thirdly, we included studies that sampled patients who were ill with COVID-19 at the time of the study, since the trials performing vitamin supplementation on healthy patients are focused on the effects of vitamin D at a preventive level, whereas, as explained above, this review focuses on treatment. Finally, the Cochrane COVID-19 Study Register is a database containing all studies that have been—and are being—conducted in relation to COVID-19. Therefore, many of the trials that are registered are unfinished (and appeared in the search results despite inserting the Report Results filter), so we thought it appropriate to include in the inclusion criteria that the studies are finished and provide the results.

### 2.3. Exclusion Criteria

The proposed exclusion criteria for this systematic review were (a) studies with insufficient data, (b) in vitro, in silico or in vivo animal studies, (c) studies using medicinal plants, (d) comments, expert opinions, case reports or letters to the editor, (e) observational studies and quasi-experiments, (f) studies in which the control group is intervened with vitamin D, and (g) patients not infected at the start of the study.

## 3. Results

The results show that in six of the trials, statistically significant differences were observed in some of the parameters analysed that were related to a better clinical outcome. The remaining five studies did not show significant differences in any of the outcomes of the variables studied. It should be noted that these five studies are also the only ones that intervene with a single dose of vitamin D, while in the rest of the studies, the intervention was carried out with a continuous regimen over time.

Findings from studies that perform vitamin D supplementation regardless of the baseline calcifediol levels of the sample at the time of trial initiation are described below.

Caballero-García et al. [30] studied the effect of treatment with cholecalciferol (2000 IU/day) for six weeks on muscle in patients with COVID-19, because muscle tissue is an important target of the inflammatory process caused after COVID-19 infection. They observed a slight improvement in the parameters assessing physical fitness and respiratory function, but it was not statistically significant. In contrast, they found a significant decrease in the circulating levels of creatine kinase (CK), a marker of muscle damage. This could suggest an immunomodulatory role of vitamin D in reducing the muscle inflammatory process that occurs in COVID-19. They further suggest that the greatest effect of vitamin D supplementation on global muscle fitness would be in individuals with suboptimal basal vitamin D levels. Circulating levels of calcifediol were significantly increased in the intervention group compared to the placebo group and to baseline levels before the intervention.

Entrenas Castillo et al. [31] suggest that the administration of high-dose calcifediol significantly reduces the need for ICU admission in patients hospitalised for COVID-19. Of 50 patients treated with vitamin D, only one required ICU admission (2%), while in the control group (*n* = 26), 13 did (50%). No statistically significant differences in the mortality rate between the two groups were reported. As for the limitations of the study, they report that it was not double-blind, they did not consider body mass index (BMI)—which is now known to be a risk factor for poor outcome in COVID-19—nor did they obtain data on calcifediol concentrations before or during the trial.

However, Elamir et al. [32] found no significant differences in the need for ICU admission between the group treated with calcitriol 0.5 μg daily for 14 days or until hospital discharge and the control group. They also found no significant differences in the duration of admission, the need for endotracheal intubation, hospital readmission or mortality. They explain that these results could be due to the small sample size (*n* = 50), and that in any case, although no statistically significant differences were shown, the numerical results indicate that treatment with calcitriol could be favourable. On the other hand, they did observe a significant change in arterial oxygen saturation (measured by the oxygen saturation/fraction of inspired oxygen index (SaO2/FiO2 index)) at discharge compared to that at admission in the group of patients who had received vitamin supplementation, so that a significant reduction in oxygen requirements was also observed in this group. The limitations were the lack of placebo and the fact that it was not a blinded study, and calcifediol levels were not measured.

Cannata-Andía et al. [33] conducted the trial with the largest number of patients in this review (*n* = 543). The intervention group received a single oral dose of 100,000 IU of cholecalciferol on the first day of hospitalisation, and follow-up was performed until discharge or death. Significant differences were obtained in the increase of serum calcifediol in the intervention group. No significant differences were observed in terms of length of hospitalisation, need for ICU admission or mortality rate between patients who received the 100,000 IU bolus vitamin D and those who did not; however, it was observed that of the total cohort group, those with higher calcifediol values on admission had less lung involvement on admission and a lower rate of ICU admissions during follow-up, both statistically significant. In contrast, no significant association was found between length of hospitalisation or mortality rate and calcifediol levels on admission. They suggest that the failure to find significant results after the administration of the 100,000 IU bolus could be explained by the lack of time that might be necessary to obtain the long-term systemic effects of calcitriol on the immune system and suggest, if so, the need to administer cholecalciferol earlier, before COVID-19 is established. In terms of limitations, they point out that the time from symptom onset to supplementation was not studied and that it was not a placebo-controlled study.

Murai et al. [34] studied a sample of 237 patients with moderate to severe COVID-19 and establish, from their results, that a single high oral dose (200, 000 IU) of cholecalciferol does not significantly reduce the duration of hospitalisation, nor does it lead to any improvement in other clinical aspects analysed (mortality, ICU admission requirement, need for mechanical ventilation and mean duration of mechanical ventilation), despite a significant increase in blood calcifediol. Therefore, they claim that the lack of clinical benefit observed in this study is independent of the ability of cholecalciferol to increase serum calcifediol levels, as a single oral dose of 200,000 IU cholecalciferol can rapidly increase these levels. In a post hoc analysis complementary to this clinical trial [35], the same authors investigated how cytokines, chemokines and growth factors associated with cytokine storm and immune imbalance were modified in patients treated with vitamin D, without finding significant changes compared to the placebo group. They explain that the time from symptom onset to cholecalciferol supplementation was relatively long (10 days on average), in addition to the time required to reach its active form, so that the time factor may have mitigated the effect of vitamin D on the inflammatory cytokine peak (which occurs first after infection, and in critically ill patients—such as the patients participating in this study—may occur at a second time point approximately between days 17 and 23 after symptom onset).

Regarding the limitations of the initial study, Murai et al. [34] refer to the relatively small sample size and the fact that the patients had several coexisting diseases and were therefore subject to different treatment regimens. Furthermore, they also add that the cholecalciferol dose was administered after a relatively long time after the onset of the first symptoms (10.3 days, on average) and, moreover, that the percentage of patients with calcifediol deficiency in the participants in this study was considerably lower than in the cohorts of other studies, possibly due to geographical differences (the study was conducted in Brazil).

Perhaps for this reason, the same authors published another study [36] based on the information collected in the first study, in which they sampled patients with severe calcifediol deficiency (<10 ng/mL). They also conclude that a single oral dose of 200,000 IU cholecalciferol does not significantly reduce the duration of hospitalisation or the other clinical aspects analysed (despite a significant increase in serum levels) in patients with moderate to severe COVID-19 and severe calcifediol deficiency, although a large heterogeneity in responses was observed, probably associated with the small sample size (*n* = 32).

Although Murai’s studies have certain limitations [34,35,36], such as late intervention with supplementation (10–11 days after admission), significant underlying disease, low prevalence of vitamin D deficiency on admission, poor matching between the intervention and control groups, and the fact that a very large bolus was given, it has been decided to include them in this meta-analysis, but their results should be taken with some caution. Some of these limitations have already been reported by Pal et al. [37].

Other studies that used cohorts of calcifediol-deficient patients to conduct the studies are detailed below.

Lakkireddy et al. [38] assume that mortality and morbidity have been shown to be high in COVID-19 patients with elevated inflammatory biomarkers (INL, CRP, LDH, IL6 and ferritin). Similarly, morbidity and mortality are elevated in patients with vitamin D deficiency. This study aimed to investigate the impact of oral vitamin D supplementation on the reduction of inflammatory markers of COVID-19, as it is known to have significant immunomodulatory potency. The study was based on a sample of patients with vitamin D deficiency (<30 ng/mL) and the intervention was performed with 60,000 IU of oral calcitriol for 8 or 10 days (depending on whether BMI <25 or >25). The difference between the two groups (vitamin supplementation vs. no supplementation) was found to be highly significant (*p* < 0.01) in the reduction of all inflammatory markers studied, with the reduction in the former group being markedly greater than in the latter. There was also a significant increase in plasma vitamin D levels in the intervention group (*p* < 0.0001). In addition, the seven patients who died during the study (intervention group *n* = 2, comparison group *n* = 5) were found to have very high levels of inflammatory markers at admission compared to the survivors, with the difference being highly significant (*p* < 0.01) for IL6, CRP and ferritin, and significant (*p* = 0.02) for INL and LDH.

Maghbooli et al. [39] conducted a study in patients hospitalised for COVID-19 with calcifediol concentrations <30 ng/mL showing a general trend towards a shorter duration of hospitalisation, need for ICU admission, need for ventilation and mortality in the group treated with 25 μg oral calcifediol daily for 60 days, but without statistically significant differences. They point out that this could be due to the need to reach optimal vitamin D levels more quickly by administering a higher dose. However, it was associated with a significant increase in the percentage of lymphocytes (*p* = 0.03) and decrease in INL (*p* = 0.02) at discharge. Lymphocytes are responsible for the specificity of the adaptive immune response and participate in host defence mechanisms against viral infections. Recent observational studies have revealed that most patients infected with COVID-19 have higher leukocyte counts and lower lymphocyte counts [40]. Furthermore, INL is considered an inflammatory marker and prognostic factor for systemic inflammation that is increased in patients with severe COVID-19. In line with these statements, this study showed a significant association between low INL levels and lower ICU admission and mortality. However, what they were unable to conclude in the study was that the decrease in INL was exclusively the effect of treatment with calcifediol, and that therefore increased plasma concentrations of calcifediol are responsible for these clinical outcomes.

Due to the high potential for COVID-19 transmission even in asymptomatic individuals, Rastogi et al. [41] wanted to investigate the effect of oral cholecalciferol supplementation on CRP negativation at day 21 in asymptomatic or mildly symptomatic individuals with low (<20 ng/mL) calcifediol levels. First, statistically significant differences in the increase in serum calcifediol levels were established (mean at day 14 was 51.7 ng/mL in the intervention group vs. 15.2 ng/mL in the non-intervention group; *p* < 0.001) and 75% of patients receiving the 60,000 IU intervention reached the target levels for this study (>50 ng/mL). Secondly, statistically significant differences were observed in CRP negativation at day 21 (62.5% vs. 20.8%; *p* < 0.018). In addition, the fibrinogen levels decreased significantly in the vitamin D-supplemented group (intergroup difference 0.7 ng/mL; *p* = 0.007). The change in fibrinogen level, although statistically significant, was modest, they explain, and may not be clinically relevant, and changes in other inflammatory markers (D-dimer, CRP and procalcitonin) were not statistically significant between the two groups. As for the limitations of the study, they report that the placebo compound did not correspond exactly in taste and texture to the vitamin formulation.

Finally, Soliman et al. [42] conducted a clinical trial in patients over 60 years of age with type 2 diabetes mellitus (DM2) and vitamin D deficiency by performing the intervention with a single intramuscular injection of vitamin D (200,000 IU). They justify the dosage of the treatment on the grounds that high-dose bolus administration has been shown to be more effective in achieving sufficient vitamin D levels than low-dose daily supplementation. After six weeks, there was no significant difference in mortality between the two groups (*p* = 0.83) or in the number of patients requiring intubation. According to the study, age, presence of hypertension and chronic obstructive pulmonary disease (COPD) were independent predictors of mortality. Notably, in this study, the mean pre-intervention vitamin D level was 10.4 ng/mL in the intervention group and 21.17 ng/mL in the control group, with statistically significant differences (p = 0.001). After the intervention, the levels were 20.54 and 21.23, respectively. This is the only study in which there is no significant difference in post-intervention vitamin D levels between the two groups. The reasons why it was decided to conduct the study with this difference in the sample are unknown. The imperfect matching between the control and intervention groups in the final Solimon et al. study is a limitation. The fact that the intervention group only achieved the same 25(OH)D3 concentrations as the control group after the intervention means that any putative effect of vitamin D in comparison to the non-supplemented group would not have been visible in this intervention population. Therefore, this study should be considered with caution.

All of the studies confirmed the safety of supplementation and no adverse effects (such as episodes of hypercalcaemia, hyperphosphataemia or kidney stones) related to supplementation were reported in any of them. Only one case of vomiting after taking cholecalciferol was recorded in a participant in one of the studies. Table 2 shows a summary of the characteristics of the reviewed studies.

On the other hand, if we look at the mean serum vitamin D levels achieved in the intervention groups in each of the studies, we obtain the results shown in Table 3.

**Figure 3:**
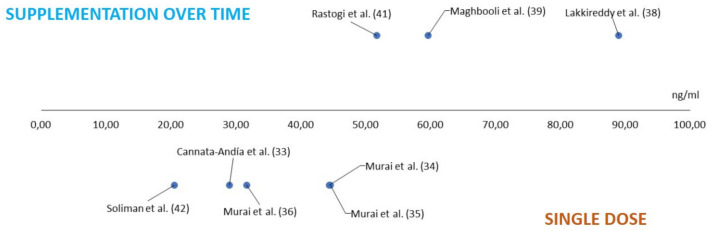
Mean plasma vitamin D levels after supplementation. Cannata-Andía et al. [33]; Murai et al. [34]; Fernandes et al. [35]; Murai et al. [36]; Lakkireddy et al. [38]; Maghbooli et al. [39]; Rastogi et al. [41]; Soliman et al. [42].

It should be noted that we do not have information on the serum levels achieved from three of the studies: Entrenas Castillo et al. [31] and Elamir et al. [32] did not collect serum vitamin D levels during the study, and Caballero-Garcia et al. [30] did not provide these data in their publication. From the information provided by the studies for which we do have such levels, we observe that in those studies that carried out supplementation over time—which are, in turn, those that observed a statistically significant improvement in at least some of the parameters analysed—higher circulating serum levels are reached after supplementation, which could explain why these studies are the ones that seem to find benefits from supplementation. In contrast, we observed that those who underwent the single-dose intervention had lower serum vitamin D levels. This correlation is shown in the graph below (Figure 3).

## 4. Discussion

There is evident heterogeneity in the results, probably due to differences in doses, duration of supplementation, parameters assessed and sample sizes. Despite this heterogeneity, it can be affirmed that most of the clinical trials analysed in this review obtained statistically significant improvements in at least one of the parameters they set out to study when vitamin D supplementation was administered to patients with COVID-19. In all of those in which these improvements were observed, the supplementation regimen was prolonged over time. Coincidentally, the trials that carried out the vitamin intervention with a single dose are the only ones in which statistically significant improvements do not appear to be observed in their results, despite finding significant increases in serum vitamin D levels in the intervention group in all of them, except in the study by Soliman et al. [42]. There is a plausible biological explanation to this phenomenon, since high-dose bolus replacement induces the expression of counter-regulatory enzymes, 24-hydroxylase and fibroblast growth factor 23 (FGF23), both of which have vitamin D-inactivating effects. On the one hand, the inactivating enzyme 1,25(OH)2D-24-hydroxylase (CYP24A1) catalyses 1,25(OH)2D3, forming 24,25-Dihydroxicholecalciferol (24,25(OH)2D) or 1,24R,25-Trihydroxyvitamin D3 (1,24,25(OH)3D), which are both largely inactive (24,25(OH)2D is the product of 25(OH)D3 catabolism by CYP24A1). On the other hand, FGF23 suppresses the 1α-hydroxylation of calcifediol, resulting in the reduced intracellular activation of vitamin D to 1,25(OH)2D3. This implies that a single high-dose bolus of vitamin D paradoxically leads to intracellular deficiency (even though the serum levels of 25(OH)D3 are risen), which could possibly explain the lack of clinical enhancement in patients receiving high-dose boluses of supplementation [43].

The secondary objective of this review was to determine whether there are differences in the effect of vitamin D supplementation according to baseline calcifediol levels at the start of the study. In this respect, and in view of the results analysed, no clear differences are observed; in other words, vitamin supplementation does not seem to have a greater or lesser impact on patients with or without vitamin D deficiency. This could be due to the immediate availability of 1,25(OH)2D3 provided through supplements to be used in immunological processes.

A randomised clinical trial to assess the efficacy and safety of vitamin D supplementation to prevent COVID-19 infection has recently been published on health care workers who acted on the front line during the pandemic [39]. This is the first controlled study to examine the role of vitamin D supplementation as a prophylactic measure to prevent COVID-19. This study could not be included in the present review because it was conducted on healthy patients (PCR-negative at the start of the study), although its results are in line with those obtained in this review. They conclude that vitamin D supplementation in highly exposed individuals prevents COVID-19 infection regardless of the baseline calcifediol status of the participants. Moreover, the protective effect was achieved at a sustained dose (4000 IU daily for one month). The increase in serum vitamin D concentrations was only moderate (8.8 ng/mL), suggesting that vitamin D intake prevents infection even if adequate serum levels are not reached [44]. In this context, it is worth noting the conclusions obtained from the very large, multi-centre study carried out by Ling et al.—which could not be included in this review due to its retrospective design—whose authors suggest finding a reduced risk of mortality in acute inpatients admitted with COVID-19 treated with cholecalciferol booster therapy, regardless of baseline serum 25 (OH)D levels [45].

SARS-CoV-2 is a recently emerged virus, and its rapid global spread has made it necessary to act quickly and design study strategies in a short time. Perhaps due to lack of time and organisation, most of the studies analysed in this review referred to a small sample size as a limitation and agree that studies on this topic need to be carried out with a larger numerical sample. In fact, on many occasions where no statistically significant results were observed, the numerical results indicated a better evolution in the group of patients who received the supplementation, which the authors possibly attributed to the small sample size. Some of the examples are given below.

In the study carried out by Caballero-García et al. [30], despite not finding statistically significant results in terms of physical condition and respiratory function, a slight improvement was observed in all of the parameters used to assess them in the intervention group. In addition, patients in this group reported feeling better. On the other hand, although Entrenas Castillo et al. [31] report no significant differences in mortality rate, none of the 50 patients treated with calcifediol died, while two of the 26 who were not treated died.

Elamir et al. [32] found no significant differences in terms of the need for ICU admission, number of days of hospitalisation, endotracheal intubation, hospital readmission or mortality, but suggest that the numerical results would favour calcitriol treatment: of the 25 patients in each arm of the trial, five required ICU admission in the vitamin D group vs. eight in the control; two vs. four, respectively, were readmitted within 30 days of discharge; no patients required endotracheal intubation or died in the interventional group, while two required intubation and three died in the control group.

In the trial performed by Murai et al. [36] in patients with severe vitamin D deficiency (<10 ng/mL), they did not obtain statistically significant results, but they still draw attention: the number of patients hospitalised <7 days was eight (out of sixteen) in the vitamin D group vs. four (out of sixteen) in the placebo group (*p* = 0.273). The mean duration of hospitalisation was 6.0 days vs. 9.5 days, respectively (*p* = 0.74). Two patients in the intervention group (12.5%) and four in the placebo group (25.0%) were admitted to the ICU during follow-up (*p* = 0.65). In the intervention group, none required mechanical ventilation and there were no deaths, while in the placebo group, one patient required mechanical ventilation and one patient died.

Maghbooli et al. [39], despite not finding statistically significant results in the fields mentioned below, found a general trend towards shorter hospitalisation time (5 vs. 6 days, on average), fewer ICU admissions (6 vs. 10 patients), less need for ventilation (two vs. five patients) and fewer deaths (three vs. five) in the vitamin D-treated group.

Finally, with the exception of one case of vomiting after taking cholecalciferol in a participant in one of the studies, there were no adverse events in any of the trials attributable to vitamin D intake, so it can be concluded that vitamin D supplementation, at least with the doses, dosages and preparations studied, is safe.

## 5. Conclusions

In view of the results discussed above, vitamin D supplementation on a daily or sustained basis is safe and could provide clinical benefit in disease progression in patients with COVID-19, irrespective of serum calcifediol levels at the time of disease acquisition. However, the results are not conclusive and clinical trials with larger sample sizes are needed to confirm this.

## Figures and Tables

**Table 1:** Inclusion criteria based on PICO algorithm.

PICO Algorithm		
P	Patient	patient with active COVID-19, regardless of severity status and serum vitamin D level
I	Intervention	with vitamin D
C	Comparison	with or without placebo plus treatment as usual
O	Results	muscle status, need for ICU admission, mortality, length of hospitalisation, inflammatory markers, arterial O_2_ saturation

**Table 2:** Characteristics of the studies reviewed.

	Sample	Methodology	Results
Caballero-García et al., 2021 [28]	30 healthy older male patients who suffered COVID-19 infection and no comorbidities Spain	Double blind study: *I* (*n* = 15): oral cholecalciferol (2000 IU/day) *C* (*n* = 15): placebo For 6 weeks from PCR+ 9 weeks	Respiratory function physical state: no statistically significant improvement ↓significant increase in circulating CK levels in I
Entrenas Castillo et al., 2020 [29]	76 patients hospitalised for SARS-CoV-2 infection Spain	Electronic randomisation: *I* (*n* = 50): Oral calcifediol (days 1, 3, 7 and weekly until discharge) *C* (*n* = 26): usual treatment Data not available	Significant reduction in the need for ICU admission in the intervention group
Elamir et al., 2022 [30]	50 patients hospitalised for SARS-CoV-2 infection USA	Open randomised trial: *I* (*n* = 25): calcitriol 0.5 µg daily for 14 days or until hospital discharge *C* (*n* = 25): usual treatment Data not available	Arterial oxygen saturation higher in I than in C at discharge compared to admission No significant changes in length of stay, ICU, endotracheal intubation, hospital readmission, or mortality
Cannata-Andía et al., 2022 [31]	543 patients hospitalised for moderate to severe COVID-19 Multicentre international. Coordinated by Spain and carried out in 12 centres from four countries (Spain, Argentina, Guatemala and Chile)	Open randomised trial: *I* (*n* = 274): single dose of 100,000 IU of cholecalciferol on the first day of hospitalisation *C* (*n* = 269): usual treatment 1 year	No statistically significant differences in length of hospital stay, ICU admissions, or mortality ↑calcifediol levels (>25 ng/mL) at admission were associated with better evolution
Murai et al., 2021 [32]	237 patients hospitalised for moderate to severe COVID-19 Multicentre, Brazil	Randomised double blind: *I* (*n* = 119): single dose of 200,000 IU of cholecalciferol *C* (*n* = 118): placebo 5 months	No significant reduction in length of hospital stay, mortality, ICU admission, or requirement or duration of mechanical ventilation, despite significantly higher calcifediol levels. ↑ in the I
Murai et al., 2022 [33]	200 patients hospitalised for moderate to severe COVID-19 Brazil	Randomised double blind: *I* (*n* = 101): single dose of 200,000 IU of cholecalciferol *C* (*n* = 99): placebo 5 months	Does not improve the status of systemic pro-inflammatory cytokines, chemokines and growth factors
Murai et al., 2021 [34]	32 patients with severe 25-OH cholecalciferol deficiency (<10 ng/mL) and moderate to severe COVID-19 disease Brazil	Randomised double blind: *I* (*n* = 16): single dose of 200,000 IU with cholecalciferol *C* (*n* = 16): placebo 5 months	No significant reduction in length of hospital stay, mortality, ICU admission, or requirement for mechanical ventilation despite significantly higher calcifediol levels. ↑ in the I
Lakkireddy et al., 2021 [36]	87 patients with hypovitaminosis (vit. D <30 ng/mL) and mild to moderate COVID-19 disease India	Open randomised trial: *I* (*n* = 44): 60,000 IU of vitamin D orally daily for 8 or 10 days depending on BMI *C* (*n* = 43): usual treatment Data not available	Significant ↓ inflammatory markers of COVID-19 (INL, CRP, LDH, IL6, Ferritin) in I
Maghbooli et al., 2021 [37]	106 patients with plasma levels of calcifediol <30 ng/mL and COVID-19 Iran	Randomised double blind: *I* (*n* = 53): 25 µg oral calcifediol daily *C* (*n* = 53): usual treatment 5 months	↑ significant of the % lymphocyte and INL ↓ ↓ INL significantly associated with ↓ length of ICU stay and ↓ mortality
Rastogi et al., 2020 [39]	40 patients with vitamin D deficiency (calcifediol <20 ng/mL) and asymptomatic or mildly symptomatic COVID-19 infection India	Randomised study: *I* (*n* = 16): 60,000 IU cholecalciferol orally for 7 days *C* (*n* = 24): placebo Data not available	↑ 41.7% in the PCR negative on day 21 in I Significant ↓ of fibrinogen levels in I, without significant changes in D-dimer, procalcitonin and CRP
Soliman et al., 2021 [40]	56 patients >60 years old with DM2 and vitamin D deficiency (<20 ng/mL) with COVID-19 Egypt	Randomised study: *I* (*n* = 40): single IM injection (200,000 IU) of cholecalciferol *C* (*n* = 16): single IM injection with placebo 2 months	No significant differences in mortality or in the need for intubation 6 weeks after diagnosis

I: intervention group; C: control group; PCR: polymerase chain reaction; CRP: C-reactive protein; CK: creatine kinase; ICU: intensive care unit; COVID-19: coronavirus disease 2019; IU: international units; BMI: body mass index; NLI: neutrophil/lymphocyte ratio; LDH: lactate dehydrogenase; DM2: diabetes mellitus type 2; IM: intramuscular.

**Table 3:** Mean plasma vitamin D level after supplementation in the intervention group.

	Mean Serum Vitamin D Level after Supplementation (ng/mL)
Cannata-Andía et al. [33]	29.00
Murai et al. [34]	44.40
Murai et al. [35]	44.60
Murai et al. [36]	31.70
Lakkireddy et al. [38]	89.00
Maghbooli et al. [39]	59.60
Rastogi et al. [41]	51.70
Soliman et al. [42]	20.54
